# Eugenol from *Syzygium aromaticum* enhances longevity and proteostasis in aged yeast

**DOI:** 10.1007/s10522-026-10448-7

**Published:** 2026-05-16

**Authors:** Suchanya Suesattayapirom, Tachaporn Kanhachai, Anjana Puttapong, Onnicha Pongwattanakewin, Phannarai Prapussorn, Yaowarin Nakornpakdee, Laran T. Jensen, Amornrat Naranuntarat Jensen

**Affiliations:** 1 Department of Pathobiology, Faculty of Science, Mahidol University, Bangkok, 10400 Thailand; 2 Biomedical Science Program, Faculty of Science, Mahidol University, Bangkok, 10400 Thailand; 3 Department of Biochemistry, Faculty of Science, Mahidol University, Bangkok, 10400 Thailand

**Keywords:** *Syzygium aromaticum* (clove), Eugenol, *Saccharomyces cerevisiae*, Oxidative stress, Anti-aging, Proteostasis

## Abstract

Clove (*Syzygium aromaticum*) extracts promote longevity in several model systems, yet the underlying molecular mechanisms responsible for the pro-longevity remain poorly defined. This study utilized a *Saccharomyces cerevisiae* model to investigate how clove extracts modulate two primary hallmarks of cellular aging: oxidative damage and the decline of protein quality control systems. Clove extracts promoted increased chronological lifespan (CLS) of yeast cells. The change in longevity was associated with a reduction in both reactive oxygen species (ROS) levels and increased resistance to oxidant and thermal challenges. The appearance of protein aggregates was also limited with treatment with clove extract and is likely linked to induction of the autophagy pathway. Deletion of *RAS2* abrogated the enhanced CLS from clove extracts. This observation suggests that the ability of clove extracts to extend the lifespan of *S. cerevisiae* is dependent on the Ras/PKA (Protein Kinase A) signaling pathway.These results indicate that the enhanced CLS from clove extracts are mediated through improving the maintenance of proteostasis rather than general antioxidant activity. Chemical profiling and comparative bioassays of major constituents identified eugenol as the principal bioactive compound, which successfully replicated the anti-aging and stress-reducing properties of clove extract. Our findings demonstrate that clove extracts, through the bioactive compound eugenol, promote survival in aged cells through reducing oxidative stress and damage and improving the clearance of aggregated proteins to limit proteotoxicity.

**Supplementary Information:**

The online version contains supplementary material available at 10.1007/s10522-026-10448-7.

## Introduction

Population aging, generally defined as the increase in people aged 60 years or older, is expected to double by 2050 (Khan et al. 2024). A major factor in this shift in population demographics is increased lifespans (Chang et al. 2025). Although average lifespans have been extended, the proportion of life in good health, referred to as healthspan, has not significantly changed (Garmany and Terzic 2024). This indicates that the additional years of life are often in poor health. The decline in life quality and the onset of numerous chronic diseases, including cardiovascular disorders, neurodegenerative diseases, cancer, diabetes, and chronic respiratory disease (Lopez-Otin et al. 2013) often become more significant in aged people.

Among the theories proposed to explain the biological mechanisms of aging, the oxidative stress theory predicts that progression of age-related characteristics are promoted by an imbalance between the production of reactive oxygen species (ROS) and the ability of antioxidant defenses to protect against the resulting damage (Golden et al. 2002; Zuo et al. 2015). While lipids, DNA, and carbohydrates can be damaged by oxidative and other stress, the abundance of proteins and the high reactivity of amino acid residues result in proteins being the primary target of oxidative damage in cells (Davies 2005). Based on this observation, it is not surprising that a hallmark of cellular aging due to ROS is the progressive decline in the ability of cells to maintain proteostasis (Hipp et al. 2019; Lopez-Otin et al. 2013). Protein damage from oxidative stress is thought to not simply be a symptom, but one of the primary factors driving cellular aging. Loss of protein function due to oxidation of side chains combined with the accumulation of damaged proteins can contribute to loss of proteostasis (David 2012; Hipp et al. 2019; Klaips et al. 2017). The decline in the ability of cells to clear misfolded proteins as they age can lead to even greater proteotoxicity and potentiate the development of age-related diseases, including Alzheimer’s, Parkinson’s, and Huntington’s disease (Liu et al. 2017; Tan et al. 2018). In addition, the failure of cells to repair or degrade oxidatively damaged proteins can lead to the formation of insoluble protein aggregates. The accumulation of protein aggregates is implicated in driving the aging process (Andersson et al. 2013; López-Otín et al. 2023) and contributes to shortened lifespans in model systems.

Although aging is an inevitable process, accumulating evidence suggests that anti-aging interventions may promote healthy longevity by delaying aging or preventing the progression of age-related diseases. It is increasingly recognized that treatments that reduce oxidative stress and the resulting protein damage can promote longevity and limit the development age-associated cellular phenotypes (Belinha et al. 2007; Kyryakov et al. 2012; Wong et al. 2009). Similarly, minimizing protein aggregation has been shown to improve cellular health and functionality (Kyryakov et al. 2012). Notably, plant extract or plant-derived natural compounds with antioxidant activity have shown potential to protect against oxidative stress and promote proteostasis, thereby slowing the aging process (Belinha et al. 2007; Dakik et al. 2020; Guo et al. 2019; Stirpe et al. 2017; Tungmunnithum et al. 2020).

An interesting plant material with anti-aging potential is the flower pods from *Syzygium aromaticum* (L.) Merr. and L.M.Perry (Merrill and Perry 1939)*,* commonly known as the spice clove. In addition to its role as a spice, clove has been utilized extensively in both Ayurveda and Traditional Chinese Medicine (Batiha et al. 2020; Devkota et al. 2022; Gil et al. 2025). Cloves are among the richest plant sources of phenolic compounds, which contribute to their strong antioxidant activity (Cortés-Rojas et al. 2014). Clove extract has demonstrated anti-aging properties in both UVB-irradiated human dermal fibroblasts and hairless mice by attenuating photodamage through an activation of the oxidative stress response pathway (Hwang et al. 2018). Furthermore, several studies have reported lifespan extension in yeast treated with clove extract (Anwar et al. 2021; Ariybah et al. 2021; Astuti et al. 2019; Fauzya et al. 2019; Lesmana et al. 2021). Despite these promising findings, the precise mechanisms underlying the anti-aging effects of clove extract and the bioactive compound responsible are not well defined.

In this study, we evaluated the potential mechanism of lyophilized clove extract to promote chronological lifespan (CLS) in yeast. Limiting both oxidative stress and damage as well as protein aggregation appeared to be key events in promoting CLS. In addition, the Ras/PKA pathway was necessary for clove extracts to effect and increase in CLS, suggesting activation of Ras2 can promote cellular survival under certain conditions. We also identified eugenol as the key bioactive compound responsible for the protective effects of clove extract. Our findings provide new insight into the anti-aging mechanisms of clove extract and eugenol, supporting their potential to promote CLS.

## Materials and methods

### Preparation of clove extracts

Clove bud powder was purchased from United Progress (Thailand) Ltd. (Bangkok, Thailand), Product code CLO019P, Lot number CLOP210665. Small-scale preparations of clove extract utilized 160 g clove powder and pilot-scale preparations used 40 kg of clove powder. Clove powder was macerated in 95% ethanol for 48 h. The resulting clove/ethanol mixture was filtered and concentrated using a Savant SVC100 SpeedVac equipped with a RT100 refrigerated condensation trap (Thermo Fisher Scientific, Waltham, MA, USA) for small-scale preparations and a model R-1050 rotary evaporator equipped with low temperature coolant circulation pump (Henan Lanphan Industry Co., Ltd., Zhengzhou, China) for the pilot-scale preparation. The clove extract from the pilot-scale preparation was dried using a Vacuum Freeze Dryer 50SM (Grisrianthong Co., Ltd., Ratchburi, Thailand). The final, powdered product was stored at room temperature in a dry location. For subsequent use in analyses, the lyophilized clove preparation was reconstituted in DMSO and stored at −20 ˚C.

### Yeast strains, plasmids, and culture conditions

The yeast *Saccharomyces cerevisiae* was first identified by Meyen (Meyen 1839). The wild-type strain *S. cerevisiae* utilized in this study was BY4742 (*MATα* *leu2∆0* *lys2∆0* *ura3∆0* *his3∆1*) (Brachmann et al. 1998). Deletion strains *tor1*∆, *sir2*∆, and *ras2*∆ were obtained from Open Biosystems (Layafette, CO, USA). Plasmid pCC013 expressing a GFP-Atg8p fusion using the *TPI1* promoter was generated by PCR amplifying *ATG8* sequences (−1 to + 354) with the introduction of 5’ BamHI and 3’ XbaI sites followed by digestion and ligation into plasmid pWC003, a variant of GFP-SMF1 (*URA3*, *CEN*) (Jensen et al. 2019; Nikko et al. 2008). Plasmid pHSP104-GFP was provided by David J. Eide and has been previously described (MacDiarmid et al. 2013). Chronological lifespan assays were performed as previously described with some modification (Hu et al. 2013). A single colony was inoculated into synthetic complete (SC) media and grown overnight at 30 °C with shaking at 220 rpm. The overnight culture was diluted into fresh SC medium to an OD_600_ of 0.1 and supplemented with the indicated concentrations of clove extract and incubated with shaking for 3 days to reach stationary phase, this was defined as Day 0 and 100% survival. CLS was monitored for indicated number of days with colony forming units (CFU) determined every 3 days after the established Day 0. Samples of the aging cultures were plated onto YPD agar and CFUs were counted. The percentage of cell survival in aging culture was calculated relative to Day 0.

### Measurement of intracellular ROS levels

2,7-Dichlorofluorescein diacetate (DCFH-DA) (Sigma, St. Louis, MO, USA) was used to measure intracellular ROS levels. Yeasts were treated with vehicle (DMSO) or clove extract and cultured as described for the CLS assay. Aliquots of cultures were removed every 6 days for ROS determination. Yeast were incubated with 10 μM DCFH-DA for 1 h. Following incubation, the samples were washed twice with phosphate buffered saline (PBS, pH 7.4) using an Eppendorf Centrifuge 5804 R (Eppendorf AG, Hamburg, Germany) at 13,200 rpm for 5 min at 25 ˚C. Cell lysates were prepared in 10 mM Na_2_HPO_4_, 5 mM EDTA, 5 mM EGTA, 50 mM NaCl, 0.1% Triton-X, 300 mM Sorbitol, 20 mM HEPES (pH 7.4), with 1% proteinase inhibitor cocktail III (Merck Millipore, Burlington, MA, USA) and 5 mM phenylmethylsulfonyl fluoride (Sigma, St. Louis, MO, USA) using glass bead extraction (Jazwinski 1990). Lysates were collected using an Eppendorf Centrifuge 5804 R at 13,200 rpm for 10 min at 4 °C. Samples were filtered using Spin-X Centrifuge Tube Filters, 0.22 µm pore size (Costar, Corning Life Sciences, Corning, NY, USA). Fluorescence intensity in clarified lysates was monitored with a Spark 10 M multimode microplate reader (Tecan Group, Ltd., Männedorf, Switzerland) using 96-well black plates, excitation at 490 nm and emission at 535 nm. Results were normalized to the protein concentration of each sample determined using the Bradford Protein Assay reagent (Bio-Rad Laboratories Inc., Hercules, CA, USA) with bovine serum albumin (BSA) as a standard (Bradford 1976).

### Assessment of oxidative and thermal stress resistance

Semi-quantitative assessment of resistance to oxidative or thermal challenges was performed using serial dilution assays (spot tests) (Astuti et al. 2019; Burstein et al. 2012; Dakik et al. 2020; Fauzya et al. 2019; Kanprasoet et al. 2015). Chronologically aging yeast cultures treated with or without clove extract were sampled on days 0, 3, and 6. Cell density was measured at optical density (OD_600_) using a Genesys 20 visible spectrophotometer (Thermo Scientific, Waltham, MA, USA). The value of 1 OD600 equivalent to 10^7^ cells/mL was used to calculate cell density. Yeast cultures were diluted with sterile dH_2_O to allow the experiments to be performed using a controlled initial cell density at 10^7^ cells/mL. Ten-fold serial dilutions were then prepared, and 10 µL aliquots were spotted directly onto YPD plates. The resulting yeast spots contained 10^5^, 10^4^, 10^3^, 10^2^, and 10 cells. For analysis of oxidative stress resistance, yeast were spotted onto YPD plates containing either 2.5 or 5 mM hydrogen peroxide (H₂O₂). For determination of thermal stress resistance, cells were spotted onto YPD plates and subjected to a heat shock at 55 ˚C for 1.5 or 2 h or incubated at 30 ˚C as a control. Following the heat shock, plates were transferred to 30 ˚C. All plates were incubated for 3 days at 30 ˚C and photographed using a ChemiDoc XRS + Imaging System (Bio-Rad, Hercules, CA, USA).

### Measurement of protein carbonylation

Protein carbonyl content was detected following derivatization with 2, 4 dinitrophenylhydrazine (DNPH) as previously described (Levine et al. 1990). Yeast cell lysates were prepared from cultures grown in SC medium treated with vehicle or clove extracts for 3 days, Day 0 in stationary phase. Protein concentration was determined using Bradford Protein Assay reagent (Bio-Rad Laboratories Inc., Hercules, CA, USA) with bovine serum albumin (BSA) as a standard (Bradford 1976). For each sample, 15 to 20 µg protein was reacted with DNPH for 15 min at 25 ˚C before the addition of a neutralization solution to terminate the reaction. DNP-derivatized proteins were resolved by 14% SDS-PAGE gel and subsequently transferred to a nitrocellulose membrane (Amersham Biosciences, Buckinghamshire, UK). Protein carbonylation was detected using an anti-DNP antibody (Merck Millipore, Burlington, MA, USA) at a dilution of 1:5000. Pgk1p was detected as a loading control using an anti-Pgk1p antibody (Abcam, Cambridge, UK) at a dilution of 1:500. GFP was detected using an anti-GFP antibody (Santa Cruz Biotechnology, Dallas, TX, USA) at a dilution of 1:5000. Visualization of immunoblots was performed using an HRP-conjugated secondary antibody and ECL detection (Merck Millipore, Burlington, MA, USA) with a ChemiDoc XRS + Imaging System. Densitometric quantitation was performed using Image Lab Software (Bio-Rad, Hercules, USA).

### Measurement of protein aggregation

Protein aggregation was detected by monitoring the localization of a GFP-tagged Hsp104p construct (MacDiarmid et al. 2013). Transformed cells were cultured in SC medium lacking uracil (SC–Ura) and treated with either DMSO (vehicle) or clove extracts. GFP fluorescence was visualized at Days 0, 3, and 6 with at least 300 cells examined for each sample. Live yeast were immobilized on agarose pads, and Hsp104p-GFP localization was monitored using a Nikon ECLIPSE 80i microscope (Nikon, Shinagawa, Tokyo, Japan), and images were captured with NIS-Elements software (NIS-Elements BR Ver. 5.41, Nikon Corporation, Japan).

### Identification and quantification of bioactive compounds

The identification and quantification of phenolic compounds from clove extract utilized high-performance liquid chromatography with a photodiode array (HPLC–PDA), as previously described (Shan et al. 2005) with minor modifications. The analysis was carried out on a Waters Alliance 2695 HPLC system equipped with a 996 PDA detector (Waters Corporation, Milford, MA, USA) using an Ascentis® C18 HPLC column (250 × 4.6 mm, 5 μm) coupled with an Ascentis® C18 SupelGuard guard cartridge (20 × 4.6 mm, 5 μm) supplied by Merck Inc. (Darmstadt, Germany). A gradient elution method was employed using mobile phase A (1.25% formic acid in type I water) and mobile phase B (100% methanol) at flow rate 0.8 mL/min. The gradient system was as follows: 95% A, 5% B (0-15 min); 70% A, 30% B (15-40 min); 60% A, 40% B (40-60 min); 50% A, 50% B (60-65 min); 45% A, 55% B (65-90 min); and finally, 100% B (90-95 min). Phenolic compounds were detected at 280 nm and 370 nm.

Standard compounds, including gallic acid hydrate, acetyl eugenol, and quercetin hydrate, were purchased from Tokyo Chemical Industry Co., Ltd. (Tokyo, Japan). Eugenol and kaempferol were obtained from Sigma-Aldrich (St. Louis, MO, USA). Stock solutions of each standard compound were individually prepared in DMSO. A mixed standard solution was prepared by combining stock solutions, followed by serial two-fold dilutions in HPLC-grade methanol to establish a calibration range. The lyophilized clove extract was dissolved in DMSO and subsequently diluted with HPLC-grade methanol to an appropriate concentration for analysis. Prior to injection, the clove extract was passed through a 0.45 μm nylon FilTrex syringe filter (Science Integration Co., Ltd., Bangkok, Thailand). Linear calibration curves for each standard compound were generated by plotting peak area versus concentration.

### Statistical analysis

Experimental data are presented as mean ± standard deviation (SD), except for lifespan assays, which are presented as mean ± standard error of the mean (SEM). Statistical analyses were performed using GraphPad Prism version 9 (GraphPad Software, San Diego, CA, USA). Differences between groups were assessed using one-way ANOVA with post-hoc Tukey's test or with Dunnett’s test.

## Results

### Clove extracts enhance chronological lifespan

The extraction method can impact the phytochemical composition and bioactivities of clove extracts (Hwang et al. 2018; Lesmana et al. 2021; Shan et al. 2005; Zhang et al. 2021). Previous studies have reported that ethanol-derived clove extract extends the chronological lifespan (CLS) of yeast cells, with the extract typically presented in a paste form following rotary evaporation from lab-scale extractions (Ariybah et al. 2021; Astuti et al. 2019; Fauzya et al. 2019; Lesmana et al. 2021). Our initial investigation utilized small-scale preparations of clove extracts. Yeast treated with the small-scale extracts exhibited a reproducible increase in CLS for two separate preparations (Fig. S1). However, powdered extracts are preferred for many applications due to their longer shelf life and ease of use in subsequent formulations (Das et al. 2014; Molnar et al. 2021). Pilot-scale preparations were prepared in order to utilize lyophilization to obtain clove extract as a powder.

**Fig. 1:**
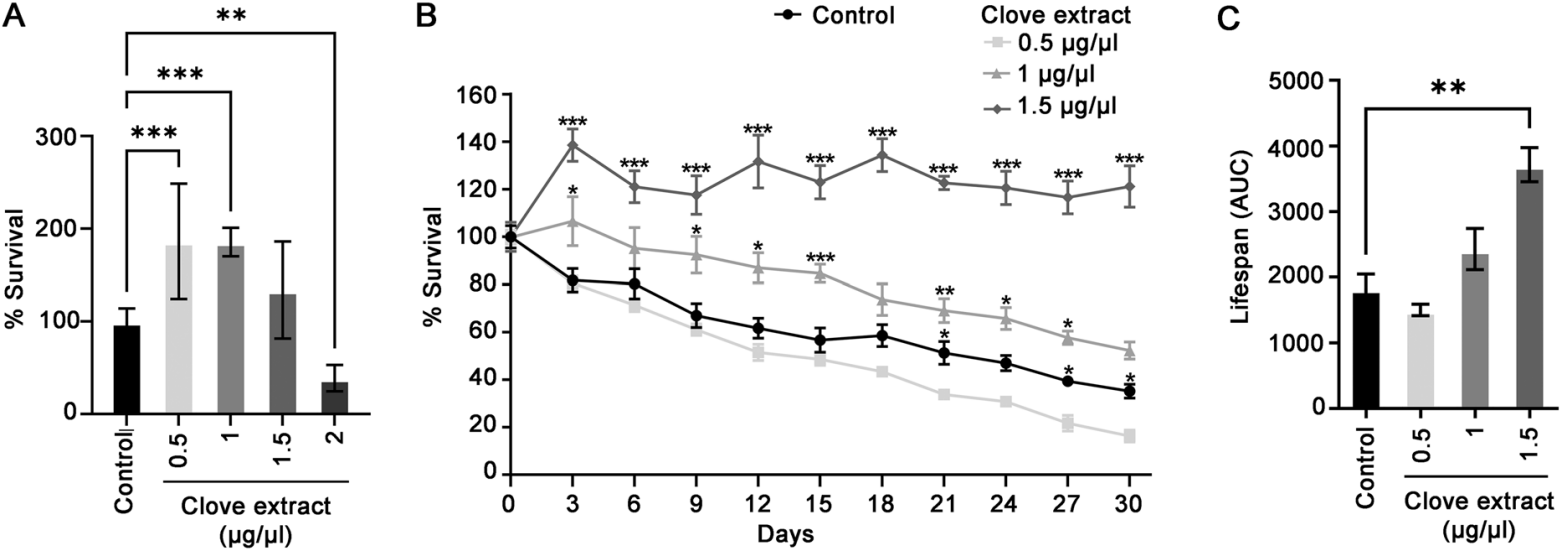
Lifespan-extending effect of large-scale prepared clove extracts in yeast. **A** Yeast cells were supplemented with indicated concentration of clove extract and grown for three days to evaluate toxicity. **B** The CLS assay was used to assess effects on longevity of the indicated concentrations of clove extracts. Every three days, aliquots of the aged cultures were plated onto YPD agar and colony forming units (CFU) were determined. **C** The area under survival curves (AUC) was determined and used to illustrate survival of each treatment over the time-period indicated. Results are from three independent experiments; values are the mean ± SEM. Statistical differences determined using one-way ANOVA with Dunnett’s post-hoc test; *P < 0.05, **P < 0.01, ***P < 0.001

Yeast were treated with pilot-scale clove extracts and incubated for three days, a growth time resulting in entry into stationary phase. Cytotoxicity of the clove extract powder was not significant at concentrations of 1.5 µg/µL or lower (Fig. 1A). Lyophilized clove extracts at 1.5 µg/µL increased CLS by 100% of control (Fig. 1B and C). These results suggest that the pilot-scale prepared clove extracts retain anti-aging properties and can significantly extend the chronological lifespan of yeast cells.

### Clove extracts limit ROS production and improve oxidative stress resistance in chronologically aged yeast

**Fig. 2:**
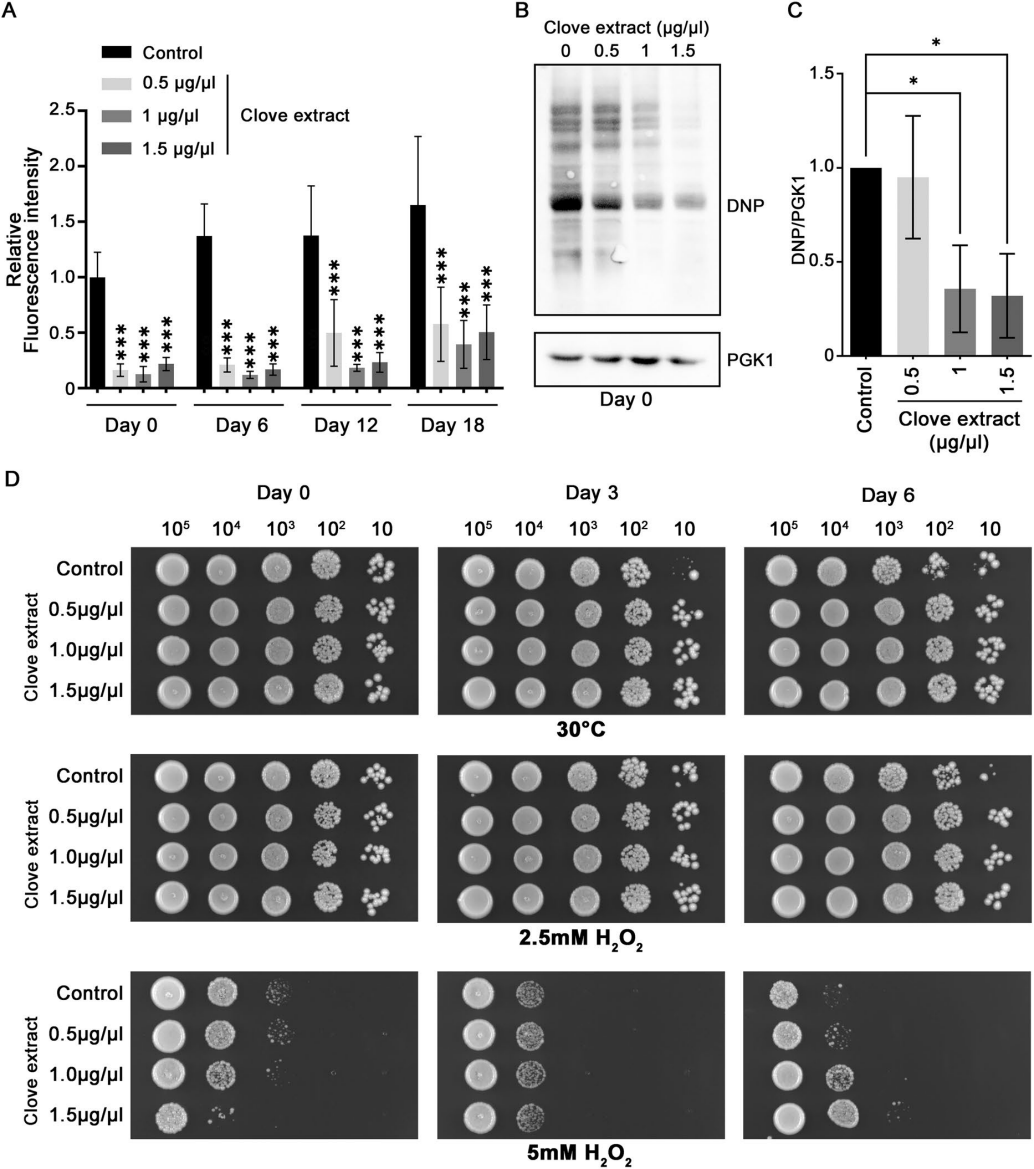
Clove extracts reduce oxidative stress and damage in chronologically aged yeast. Yeast cultures were treated with the indicated concentrations of clove extract or vehicle control. **A** Intracellular ROS levels were measured using the fluorescent probe DCFH-DA in aging cultures collected every 6 days. Fluorescence intensity was normalized to total protein concentration, with the vehicle control at Day 0 set to 1. Statistical significance was determined using one-way ANOVA followed by Tukey’s post-hoc test; ***P<0.001. **B** Protein carbonylation, a marker of oxidative protein damage, was assessed with derivatization of protein samples with 2,4-dinitrophenylhydrazine (DNPH) followed by detection using immunoblots. Pgk1p was monitored as a loading control. **C** Quantification of protein carbonylation immunoblots normalized to the Pgk1p content. The content of carbonylated protein in vehicle control was set to 1. Statistical significance was determined using one-way ANOVA followed by Dunnett’s post-hoc test; *P<0.05. **D** Oxidative stress tolerance was evaluated by spotting tenfold serial dilutions of aging cultures onto YPD agar plates supplemented with hydrogen peroxide (H_2_O_2_) at the indicated concentrations. Plates were imaged after 3 days of incubation. Data are mean ± SD from three independent experiments

Clove extract treatment resulted in a significant and sustained reduction in intracellular ROS levels. Treatment with clove extract at all concentrations (0.5, 1, and 1.5 µg/µL) resulted in ROS levels approximately 10% to 20% of untreated cells at Day 0. The reduced ROS content from clove treatment was evident throughout the experiment. At Day 18 in stationary phase, the last day examined, ROS levels remained reduced in cells treated with lyophilized pilot-scale clove extract, at approximately 35% to 40% of untreated cells (Fig. 2A).

Protein oxidation can occur under conditions of elevated ROS levels, resulting in irreversible protein damage. The impact of clove extract on protein oxidation was evaluated by monitoring protein carbonylation, a marker of oxidative protein damage induced by ROS (Fedorova et al. 2014). At Day 0 in stationary phase (3 days in culture), exposure to 1 and 1.5 µg/µL clove extract caused a substantial reduction in the accumulation of protein oxidation. However, treatment with 0.5 µg/µL clove extract did not result in a significant reduction in protein carbonyls (Fig. 2B and C). While ROS levels are reduced by 0.5 µg/µL clove extract, it is possible that this concentration is below the threshold for limiting protein damage. It was not possible to obtain data for protein carbonylation at subsequent days in the CLS experiment as protein content of yeast extracts was not sufficient to perform the analysis.

Enhanced resistance to oxidative stress was also evident in aged cells. Exposure to clove extracts, at all concentrations tested, reversed impaired growth of yeast challenged with 2.5 mM H_2_O_2_. Treatment with 1 and 1.5 µg/μL clove extract was protective against 5 mM H_2_O_2_ in aged yeast (assayed at 6 days in stationary phase) (Fig. 2D). The pro-longevity effec﻿t of clove extracts appears not to be limited to actively growing cells and provides protection, limiting ROS production and oxidative stress in aged cells.

### Clove extracts protect against protein aggregation and thermal stress in aged yeast cells

**Fig. 3:**
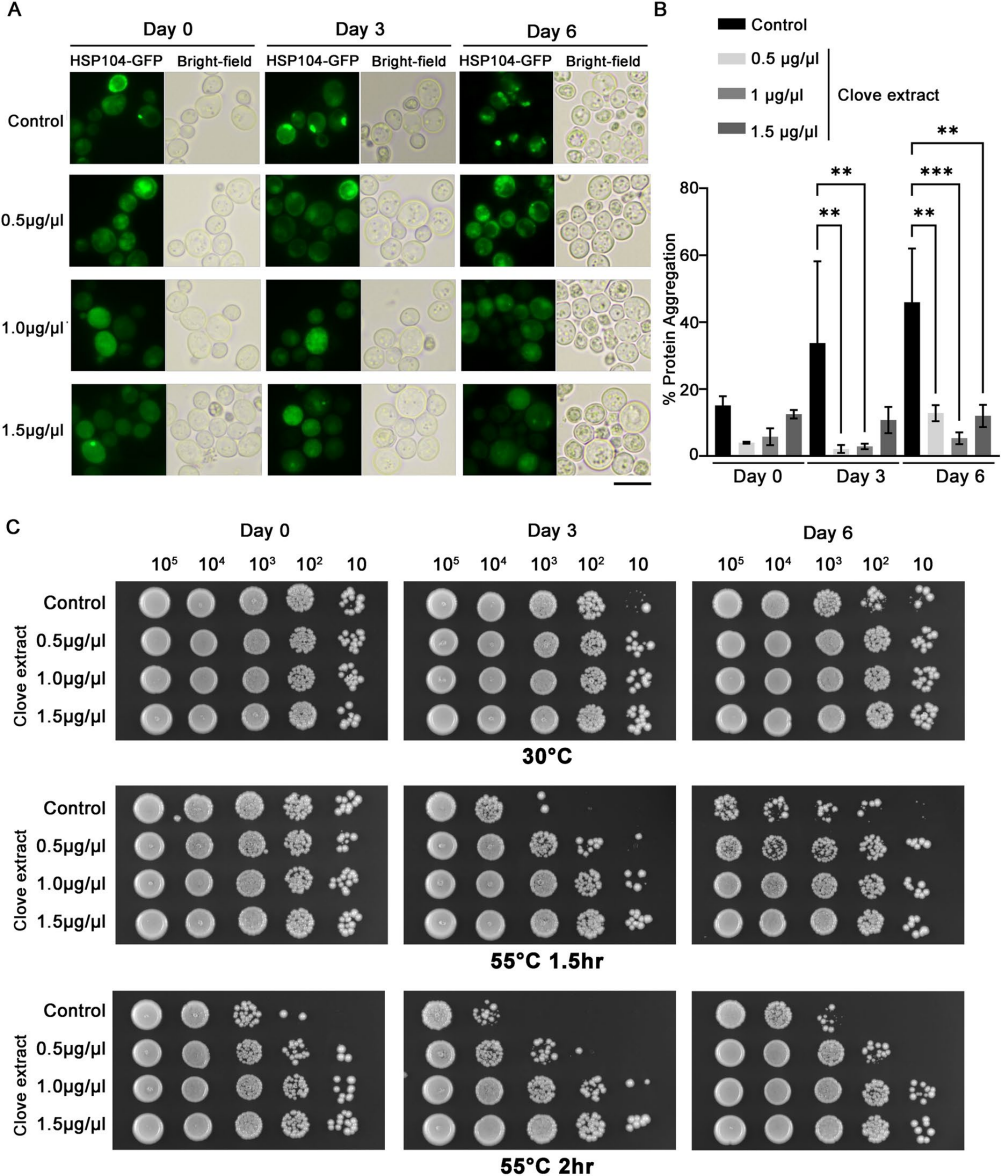
Clove extract limits appearance of protein aggregates and enhances thermal stress resistance in chronologically aged yeast cells. **A** Aggregated proteins were visualized by monitoring Hsp104-GFP loci at the indicated days of aging. **B** The percentage of cells containing aggregates was determined using at least 300 cells for each condition. **C** Thermal sensitivity was assessed in aged yeasts following exposure to 55 °C for 1.5 and 2 h. Cells were spotted onto YPD plates and grown for 30 ˚C for 3 days. Data are the mean ± SD from three independent experiments. Statistical differences between control and clove treated samples were determined using a one-way ANOVA with Tukey’s post-hoc test; **P < 0.01 and ***P<0.001

Oxidative stress can promote protein aggregation, as physical damage to proteins can lead to unfolding and exposure of the hydrophobic core. Protein aggregation during chronological aging was monitored using the localization of Hsp104p-GFP. Hsp104p-GFP colocalizes with aggregated proteins, allowing the visualization of protein aggregates as GFP foci (Erjavec et al. 2007). Protein aggregation was observed in untreated yeast at Day 0 in stationary phase, and the number of cells exhibiting GFP foci showed a statistically significant increase on Day 6. In contrast, substantial numbers of GFP foci were not evident in aged cells treated with all concentrations of lyophilized clove extract. At Day 0 the number of cells showing GFP foci was reduced approximately fivefold in cells treated with 0.5 and 1 µg/µL clove extract. Although the results for 1.5 µg/µL clove extract on Day 0 showed an approximately twofold decrease, the result did not reach statistical significance. However, on Day 6, yeast treated with clove extracts at all concentrations exhibited a significant decrease in GFP foci compared to untreated cells (Fig. 3A and B).

Thermotolerance is linked to proteostasis and elevated temperature is a potent stress that disrupts protein folding and stability (Andersson et al. 2013). In addition, thermal stress directly induces ROS production which further promotes protein unfolding (Davidson and Schiestl 2001). Thermotolerance in aged cells is associated with enhanced viability under stress conditions (Burstein et al. 2012; Dakik et al. 2020; Kyryakov et al. 2012; Wei et al. 2008). Sensitivity of untreated aged cells to heat stress, 55 ˚C for 1.5 or 2 h, increased with the duration in stationary phase. Treatment with clove extracts was protective against thermal stress in aged cells. All concentrations of clove extract examined improved survival during heat stress, with higher concentrations providing more protection (Fig. 3C). These findings suggest that clove extracts may have an impact on cell aging through enhancing pathways that protect against accumulation of mis-folded or damaged proteins.

### Autophagy is induced in yeast cells by treatment with clove extract

**Fig. 4:**
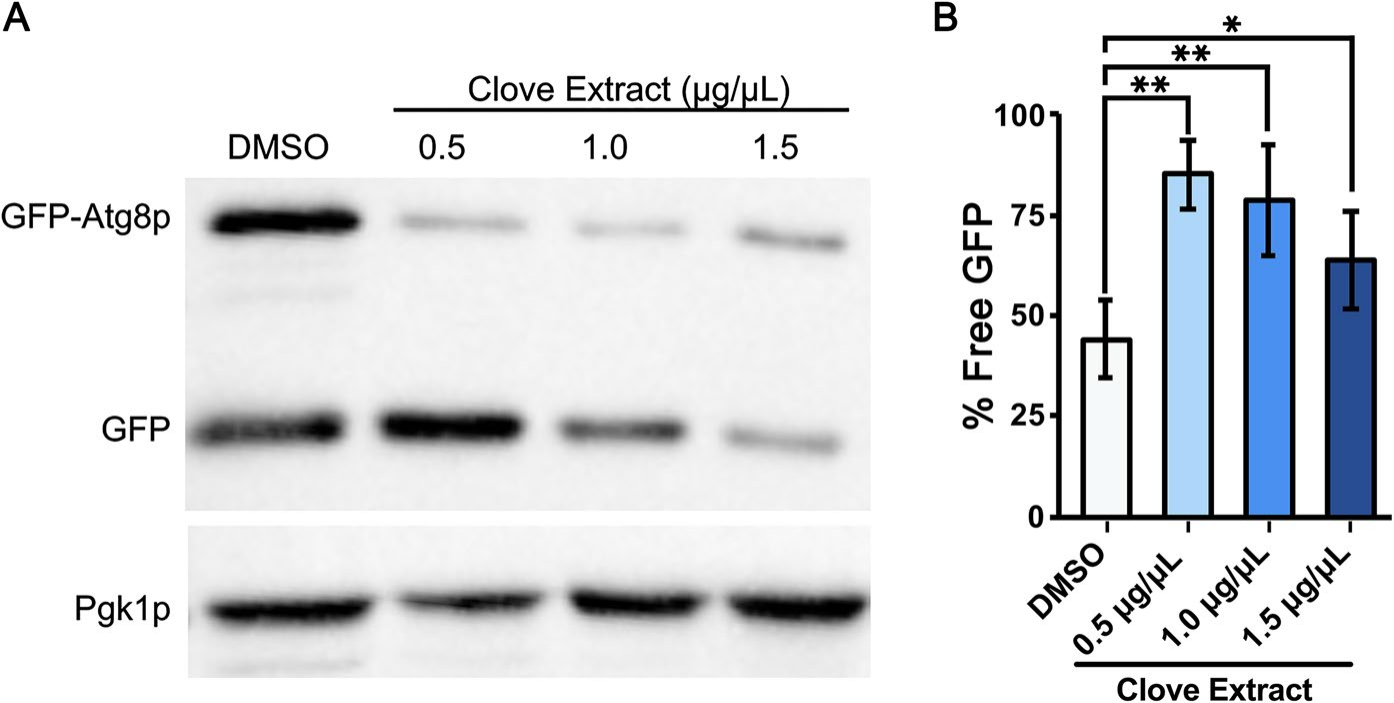
Clove extracts promote activation of autophagy. **A** WT yeast transformed with plasmid pCC013 (GFP-Atg8p) were grown in SC medium lacking uracil for three days to reach Day 0 in stationary phase. GFP was detected using immunoblots to detect GFP-Atg8p and free GFP. **B** The percent of free GFP was quantitated for each sample. Statistical differences between control and clove treated samples were determined using a one-way ANOVA with Tukey’s post-hoc test; *P < 0.05 and **P < 0.01

Autophagy is a critical cellular clearance mechanism for removing misfolded protein aggregates (Klionsky 2011). Examining autophagy activation at Day 0 using a GFP-Atg8p probe (Nair et al. 2011) revealed a significant increase in the cleavage of the GFP-Atg8p probe to produce free GFP, an indicator of autophagy activity (Fig. 4A). Yeast treated with 0.5 and 1.0 µg/µL clove extract exhibited an approximate two-fold increase in free GFP. While activation of autophagy in yeast exposed to 1.5 µg/µL clove extract was more limited, an increase of 1.4-fold relative to the DMSO vehicle control was observed (Fig. 4B). As was the case for the analysis of protein carbonyls, it was not possible to obtain sufficient protein in yeast lysates to assay autophagy at later days in the CLS assay. In any case, the reduction of age-associated accumulation of protein aggregates in yeast, and potentially enhanced survival following thermal stress appear to be linked to effects of clove extract to activate autophagy to remove protein aggregates, improving cellular survival.

### Enhanced CLS from clove extracts requires an intact Ras2 pathway

**Fig. 5:**
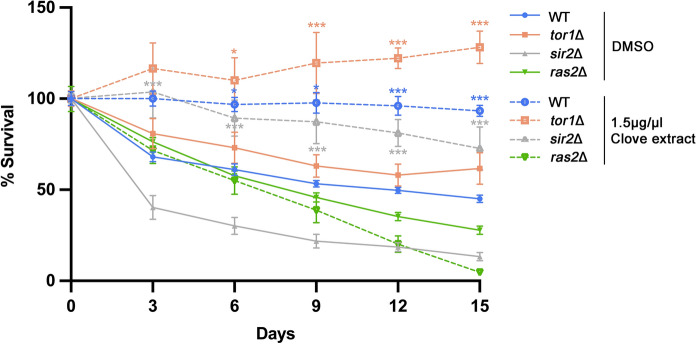
Clove extracts require an active Ras2 pathway for enhanced CLS. The CLS assay was performed as described in Fig. 1. CLS was compared between vehicle control (DMSO) and clove extract at 1.5 µg/µL in WT, *tor1*∆, *sir2*∆, and *ras2*∆ strains. Statistical differences between control and clove-treated samples were determined using a one-way ANOVA with Tukey’s post-hoc test; *P < 0.05 and ***P < 0.001

To identify the genetic pathways required for clove extract-mediated longevity, we performed chronological lifespan (CLS) assays using several key yeast signaling mutants: *ras2*Δ, *tor1*Δ, and *sir2*Δ. Our results demonstrate that the enhanced CLS from clove extract is dependent on the Ras/PKA pathway. Clove extract failed to extend the lifespan of the *ras2*Δ mutant. In contrast, clove extract significantly extended the CLS of both *tor1*Δ and *sir2*Δ mutants (Fig. 5). Interestingly, in our experimental conditions, the *ras2*Δ mutant exhibited a reduced baseline lifespan compared to wild-type cells; however, the lack of an additive or rescue effect by the extract confirms that functional Ras2 signaling is a prerequisite for its anti-aging activity. These findings indicate that the primary mode of action for clove extract is independent of the TOR and Sirtuin pathways and instead functions through the modulation of the Ras2 signaling axis.

### Identification of eugenol as the bioactive compound in clove extract that promotes yeast CLS

**Fig. 6:**
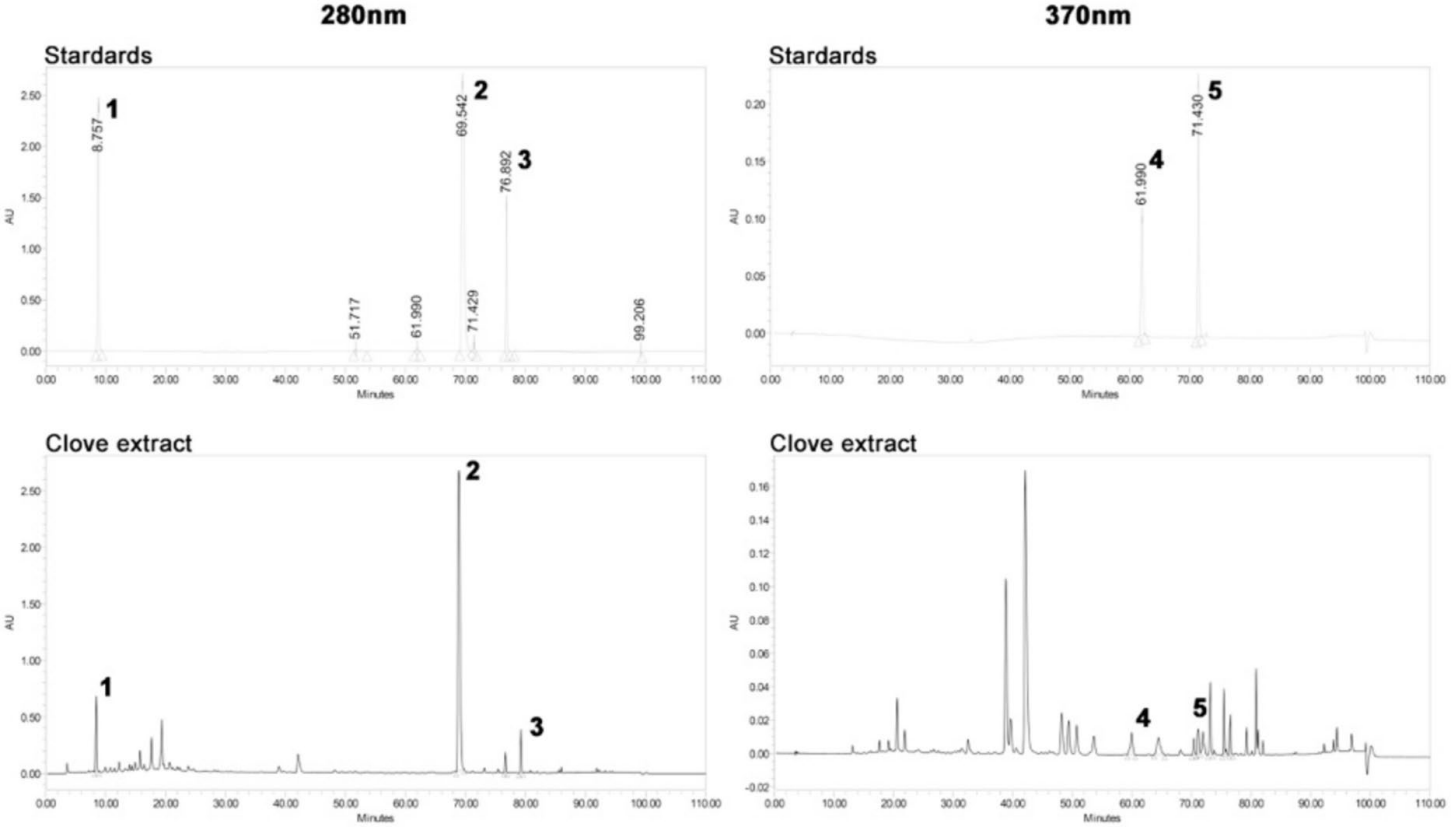
Major component analysis of clove extract. The concentration of major phenolic compounds was determined using HPLC analysis. Detection utilized 280 nm and 370 nm. Compounds identified are (1) gallic acid, (2) eugenol, (3) acetyl eugenol, (4) quercetin, and (5) kaempferol

**Fig. 7:**
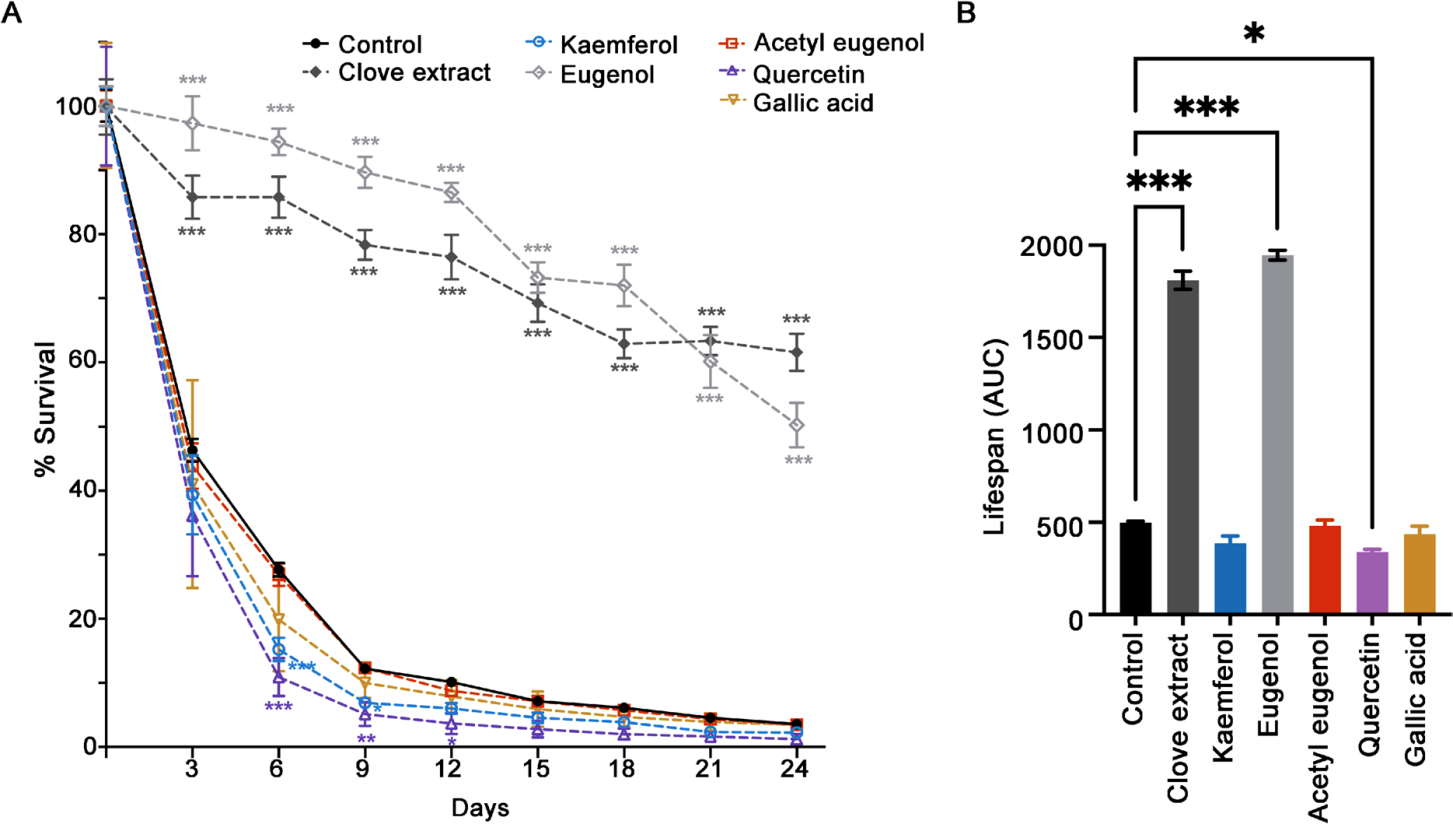
Eugenol recapitulates the CLS extending effect of clove extract. **A** The CLS assay was performed as described in Fig. 1. CLS was compared between clove extract and phenolic compounds determined at the concentration found within 1.5 µg/µL clove extract.**B** Quantification of the area under the survival curve (AUC) for each condition tested. Data are the mean ± SEM from three independent experiments. Statistical differences between control and clove treated samples were determined using a one-way ANOVA with Dunnett’s post-hoc test; *P < 0.05 and ***P < 0.001

To identify the active compound(s) in clove extract with potential for extending yeast CLS, the major phenolic compounds were determined using HPLC analysis. The HPLC profiles of lyophilized clove extracts (Fig. 6) were consistent with the presence of gallic acid (1), eugenol (2), acetyl eugenol (3), quercetin (4), and kaempferol (5). Several minor peaks in the clove extract remained unidentified. Eugenol was the most abundant phenolic constituent, followed by kaempferol, gallic acid, acetyl eugenol, and quercetin (Table 1). To investigate the bioactive potential of these components, CLS assays were performed by treating yeast cells with individual phenolic compounds at concentrations corresponding to those found in the 1.5 µg/µL clove extract. Only eugenol significantly extended lifespan of yeast cells (Fig. 7A). The extension of CLS in yeast treated with eugenol and clove extract was similar. In contrast, the other phenolic compounds identified in clove extract in this study did not extend yeast lifespan (Fig. 7B). These findings indicate that eugenol is the major active component in clove extract responsible for delaying chronological aging in yeast.

**Table 1:** Quantification of Major Phenolic Compounds in clove extract by HPLC–PDA analysis

No	Compound	µg compound/mg of clove extract
1	Gallic Acid	9.6
2	Eugenol	190
3	Acetyl Eugenol	5.0
4	Quercetin	0.4
5	Kaempferol	38

### Eugenol mimics effects of clove extracts in protecting aged cells against oxidative and thermal stress

**Fig. 8:**
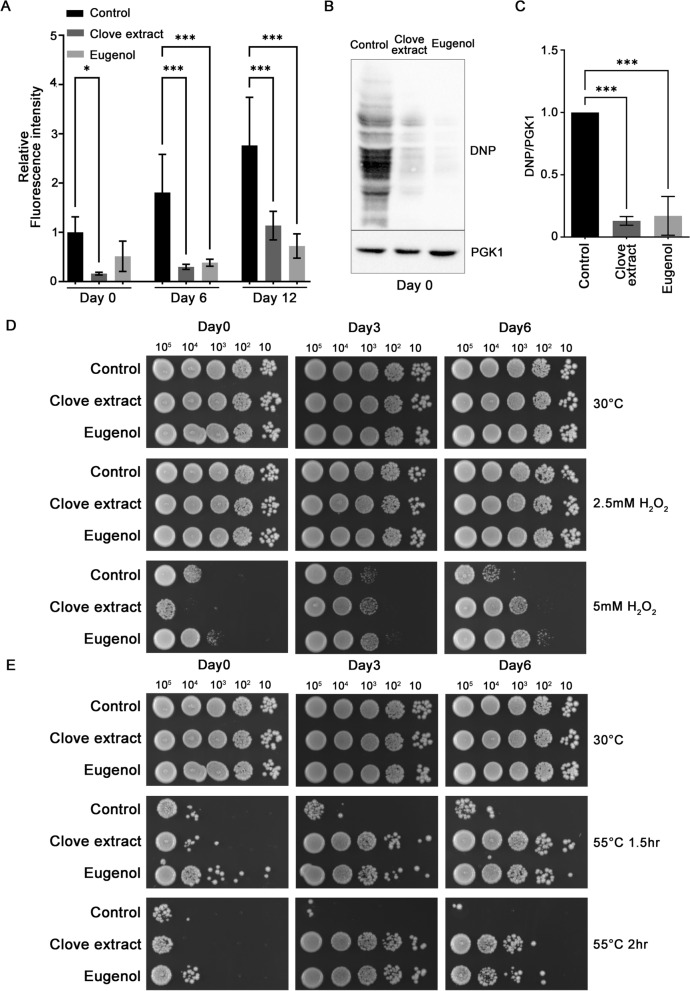
Eugenol reduces ROS and oxidative damage and enhances stress tolerance in aged yeast cells. Aged yeast treated with vehicle (DMSO), clove extracts at 1.5 µg/µL, and 0.3 µg/µL eugenol were assessed for **A** intracellular ROS using DCFH-DA. Data are the mean ± SD from at least three independent experiments. Statistical differences were determined using one-way ANOVA followed by Tukey’s post-hoc test; *P<0.05 and ***P<0.001. **B** Protein carbonylation using immunoblots and **C** quantitation of protein carbonyl levels. Data are the mean ± SD from at least three independent experiments. Statistical differences were determined using one-way ANOVA followed by Dunnett’s post-hoc test; ***P<0.001. **D** Oxidative stress resistance was assessed with H_₂_O_₂_ treatment, **E** thermotolerance to exposure to 55 ˚C for 1.5 and 2 h as described in Fig. 2 and 3

Eugenol was investigated for its ability to reduce ROS accumulation and protein carbonylation as well as to enhance resistance to oxidative and thermal stress. Clove extract and eugenol exhibited a similar ability to limit ROS in aged yeast from cultures from Day 0 to Day 18 in the stationary phase (Fig. 8A). Eugenol treatment significantly reduced carbonylated proteins in aged yeast cells, similar to the effects observed with clove extract (Fig. 8B and C). In addition, both eugenol and clove extract promoted resistance to 5 mM H_2_O_2_ and thermal stress at 55 ˚C (Fig. 8D and E). Taken together, these findings suggest that eugenol is a key active component within clove extract responsible for the reduction in intracellular ROS levels, limiting oxidative protein damage, and enhancing resistance to both oxidative and thermal stress in chronologically aged yeast cells. These effects are consistent with improved proteostasis and enhanced viability of aged cells.

## Discussion

Pharmacological interventions that slow the effects of aging are actively being pursued to better meet the needs of the aging population (Le Couteur et al. 2025; Petr et al. 2024). Clove extracts are a promising source of bioactive molecules and have been used in Ayurveda and Traditional Chinese Medicine for many applications (Batiha et al. 2020; Devkota et al. 2022; Gil et al. 2025). Clove extracts have also been reported to extend chronological lifespan of the baker’s yeast *S. cerevisiae* and nematode *C. elegans*, well-established model organisms for studying aging (Golden et al. 2002; Longo and Fabrizio 2012; Mack et al. 2018). Although beneficial effects of clove on enhancing longevity have been previously reported (Ariybah et al. 2021; Astuti et al. 2019; Fauzya et al. 2019; Lesmana et al. 2021), the cellular effects of clove extract on aged cells have not been well characterized.

Our findings offer molecular insights into how clove extract promotes lifespan extension. A threshold effect was observed on aged cells using three doses of clove extract (0.5, 1, and 1.5 µg/µL) from the pilot-scale preparations. While clove extracts at 0.5 and 1 µg/µL did not exhibit toxicity during exponential growth, no significant increase in lifespan was observed compared to the vehicle control. To enhance CLS, a concentration of clove extract (1.5 µg/µL) was required.

In contrast to results for CLS extension, the protective effects of clove extract on ROS levels and stress resistance were evident at lower concentrations. Clove extracts at all concentrations examined were effective at significantly reducing ROS content in aged cells. Similarly, clove extracts at each concentration limited the accumulation of protein aggregates in aged cells to comparable levels. However, resistance to either H_2_O_2_ or thermal stress exhibited a dose-dependent effect with greater protection as the clove extract concentration was increased. Among the parameters analyzed, oxidative damage to proteins, resulting in carbonylation, most closely matched the concentration effects seen for enhanced CLS, with protection apparent only at higher concentrations of clove extract.

Reduction of ROS levels demonstrates that even the lowest dose of clove extracts contain potent antioxidant activity. However, the lack of protection against protein carbonylation at the low dose suggests a multi-faceted mechanism. ROS are transient species and can be rapidly neutralized; the lowest dose clove extract appears to provide sufficient antioxidant capacity to eliminate ROS in aged cells. Protein aggregates are typically formed through hydrophobic interactions and reducing ROS levels in the cellular environment can prevent new aggregates from forming or allow degradative pathways to function more effectively (Hasanbašić et al. 2018). In contrast, protein carbonylation is an irreversible, relatively stable marker of oxidative damage (Fedorova et al. 2014). It is possible that higher concentrations of clove extract provide a secondary layer of protection, limiting the accumulation of carbonylated proteins.

This biphasic response is consistent with reports for the effects of ethanolic clove extract in *Schizosaccharomyces pombe*, where high doses showed protection only at later aging stages (Astuti et al. 2019; Fauzya et al. 2019; Lesmana et al. 2021). Plant extracts containing polyphenols are known to induce stress responses, such as the yeast Yap1p and Msn2p/Msn4p pathways (Görner et al. 1998; Toone and Jones 1999). These factors cooperate to reduce oxidative stress, limiting damage to proteins (Yap1p) and enhancing the abundance of molecular chaperones to promote refolding or clearance of damaged proteins (Msn2p/4p) (He and Fassler 2005). Interestingly, the activity of Msn2p/Msn4p and Yap1p regulators is coordinated by Ras2 and the PKA pathway (Uvdal and Shashkova 2023).

Interventions that boost proteostasis have been shown to extend lifespan in model organisms (Sampaio-Marques and Ludovico 2018). In addition to limiting oxidative damage to proteins and upregulating expression of molecular chaperones, improved proteostasis following treatment with clove extract appears to also be linked to autophagy activation. Clove extract has been previously reported to enhance autophagic activity in yeast (Ariybah et al. 2021). Together, these results suggest that clove extract promotes CLS not only due to its ability to act as an antioxidant but also through improved maintenance of proteostasis. Glycerol metabolism can also impact proteostasis through its function as a chemical chaperone (Upagupta et al. 2017). The effects of clove extracts on glycerol metabolism were not directly addressed in this study. However, the mechanistic data for the *ras2*∆ strain can inform regarding the contribution of glycerol on CLS under the conditions examined. When Ras2p activity is low glycerol synthesis as well as glycerol utilization is upregulated due to activation of Msn2p/Msn4p activity (Uvdal and Shashkova 2023). In our experimental conditions, deletion of *RAS2* leads to a severe impairment of survival, indicating that unregulated production and utilization of glycerol may place the *ras2*∆ strain at a competitive disadvantage. Activation of the Ras2 pathway may reduce glycerol production and allow the cells to adjust their metabolism to compensate for the aerobic conditions.

To gain insight into the pro-longevity effects of the lyophilized pilot-scale clove extract the chemical profile was determined. Five phenolic compounds, gallic acid, eugenol, acetyl eugenol, kaempferol, and quercetin were identified in our clove extract, similar to previous reports for small-scale prepared extracts (Shan et al. 2005). Each of the major constituents in the clove extracts were evaluated for effects on CLS using the concentration corresponding to the amount present in 1.5 µg/µL clove extract. This analysis was distinct compared to previous reports in that the effect of the clove extract was directly compared to the individual constituents at the equivalent concentrations.

Our findings demonstrate that treatment with eugenol alone provided similar extension of CLS to that of the clove extract. In addition, eugenol recapitulated the protective effects observed with the clove extract, including reduced ROS accumulation, decreased oxidative protein damage and aggregated protein, and improved resistance to both oxidative and thermal stress. These observations are in agreement with prior reports describing the antioxidant activity of eugenol and its ability to maintain proteostasis in diverse biological systems (Anwar et al. 2021; Hwang et al. 2018; Jung et al. 2023; Parween et al. 2022; Sharma et al. 2023). Despite the strong similarity between eugenol and the whole clove extract, we observed subtle differences in their protective activities. Specifically, eugenol conferred a greater level of protection against both oxidative and thermal stress in aged yeast, particularly during the early aging stages. The differences noted suggest the possibility of antagonistic effects or interference from minor constituents present in the complex whole extract mixture that may modulate the immediate protective action of eugenol.

Our analysis indicates that extension of CLS appears to not be associated with a simple reduction in oxidative stress or limiting of protein aggregation from clove extracts. Instead, modulation of cellular responses to stress, such as the Ras/PKA pathway, are also essential for enhanced CLS. While eugenol was the primary active component responsible for the enhanced CLS and protection against stress, we cannot exclude the potential for other constituents to contribute to the overall biological activity. The complexities inherent to phytochemical mixtures warrant further investigation to fully map the synergistic or antagonistic interactions that may occur. Overall, this work provides support for further studies on the therapeutic potential of lyophilized clove extract and eugenol in modulating age-related cellular decline and diseases linked to impaired protein homeostasis.

## Supplementary Information

Below is the link to the electronic supplementary material.

Supplementary file1 (DOCX 798 KB)

## Data Availability

Data are available from the corresponding author Amornrat N. Jensen (email: amornrat.nar@mahidol.ac.th) upon request.
