# Supplementary material for: Eugenol from Syzygium aromaticum enhances longevity and proteostasis in aged yeast
Source: Biogerontology. 2026 May 16;27(3):105. doi: 10.1007/s10522-026-10448-7 (PMC13179187; doi:10.1007/s10522-026-10448-7)
Supplement: Supplementary file 1 — Supplementary file1 (DOCX 798 KB) [file 10522_2026_10448_MOESM1_ESM.docx]

**Figure S1. Reproducibility of lab-scale clove extract preparations on yeast chronological lifespan, intracellular ROS, and protein carbonylation.** (A) Yeast chronological lifespan (CLS) was assessed to verify the consistency of independent extract preparations. Yeast cultures were treated with DMSO (vehicle control) or 0.3 μg/μL clove extract from two independent lab-scale extractions (Batch 1 and Batch 2). Aliquots were sampled every 3 days and plated on YPD agar to determine colony-forming units (CFU), with Day 0 survival set as 100%. (B) Area under the curve (AUC) analysis of the survival data shown in (A), representing the relative survival of each treatment group. (C) Intracellular ROS levels were monitored in aging cultures sampled on days 0, 6, 12, and 18 using the fluorescent probe DCFH-DA. Fluorescence intensity was normalized to total protein concentration, with the vehicle control at Day 0 set to 1.0. (D) Representative assessment of protein carbonylation levels in aging yeast. Statistical significance was determined using one-way ANOVA followed by post-hoc test; *P < 0.05, P < 0.01, ***P < 0.001.
